# Haptic Rendering of Diverse Tool-Tissue Contact Constraints During Dental Implantation Procedures

**DOI:** 10.3389/frobt.2020.00035

**Published:** 2020-03-20

**Authors:** Xiaohan Zhao, Zhuoli Zhu, Yu Cong, Yongtao Zhao, Yuru Zhang, Dangxiao Wang

**Affiliations:** ^1^Beijing Unidraw Virtual Reality Technology Research Institute Co. Ltd., Beijing, China; ^2^State Key Laboratory of Oral Disease, National Clinical Research Center for Oral Diseases, West China Hospital of Stomatology, Sichuan University, Chengdu, China; ^3^State Key Laboratory of Virtual Reality Technology and Systems, Beihang University, Beijing, China; ^4^Beijing Advanced Innovation Center for Biomedical Engineering, Beihang University, Beijing, China; ^5^Peng Cheng Laboratory, Shenzhen, China

**Keywords:** haptic rendering, dental implantation procedures, surgery simulation, contact constraints, state switching

## Abstract

Motor skill learning of dental implantation surgery is difficult for novices because it involves fine manipulation of different dental tools to fulfill a strictly pre-defined procedure. Haptics-enabled virtual reality training systems provide a promising tool for surgical skill learning. In this paper, we introduce a haptic rendering algorithm for simulating diverse tool-tissue contact constraints during dental implantation. Motion forms of an implant tool can be summarized as the high degree of freedom (H-DoF) motion and the low degree of freedom (L-DoF) motion. During the H-DoF state, the tool can move freely on bone surface and in free space with 6 DoF. While during the L-DoF state, the motion degrees are restrained due to the constraints imposed by the implant bed. We propose a state switching framework to simplify the simulation workload by rendering the H-DoF motion state and the L-DoF motion state separately, and seamless switch between the two states by defining an implant criteria as the switching judgment. We also propose the virtual constraint method to render the L-DoF motion, which are different from ordinary drilling procedures as the tools should obey different axial constraint forms including sliding, drilling, screwing and perforating. The virtual constraint method shows efficiency and accuracy in adapting to different kinds of constraint forms, and consists of three core steps, including defining the movement axis, projecting the configuration difference, and deriving the movement control ratio. The H-DoF motion on bone surface and in free space is simulated through the previously proposed virtual coupling method. Experimental results illustrated that the proposed method could simulate the 16 different phases of the complete implant procedures of the Straumann® Bone Level(BL) Implants Φ4.8–L12 mm. According to the output force curve, different contact constraints could be rendered with steady and continuous output force during the operation procedures.

## Introduction

Dental implantation refers to the process of implanting one or more implants made of artificial materials into the alveolar bone. It has been widely proved that implanted teeth can achieve similar restoration effects with natural teeth in aspects of aesthetics and function. Therefore, dental implantation has become the preferred restoration methods by more and more patients with lost teeth.

Dental implantation surgery mainly involves bone drilling to prepare the implantation bed for placing the implant. Compared with ordinary bone drilling surgeries such as cavity preparation, motor skill learning of dental implantation surgery is more difficult for novices because it involves fine manipulation of different dental tools to fulfill a strictly pre-defined procedure. Currently dental students mainly practice on plastic jaws and animal bones. The main problem of these training methods is the lack of training cases, as well as the large gap of operation feelings to live patients. In addition, the material consumption during practice will increase training cost, making it difficult for students to get sufficient training opportunities. With the development of virtual reality technology, the haptic surgery simulation, which can provide realistic visual-haptic perception, brings new opportunities for implant surgery training. Several approaches have been reported on bone drilling simulation involving haptics (Ranta and Aviles, 1999; Thomas et al., 2000; Forsslund et al., 2009; Luciano et al., 2009; Tse et al., 2010; de Boer et al., 2012; Yamaguchi et al., 2013), and many of them are related to the simulation of dental implantation.

Many works have been carried out on bone drilling in various biomedical applications (Petersik et al., 2002; Agus et al., 2003; Kim and Park, 2006; Morris et al., 2006; Acosta and Liu, 2007). These works share similarities in the simulation models, which are often the volume model due to its high efficiency in collision detection and describing topological changes (Avila and Sobierajski, 1996; Mcneely et al., 1999). However, they distinguish from each other on the haptic rendering methods. In the early stage the haptic rendering methods were mostly the penetration method (Mcneely et al., 1999) that directly computed the penetration force from the penetration depth. The main drawback of the penetration method is that they always suffer from visualized geometric penetration and instability issues. Then the virtual coupling method (Duriez et al., 2008) and the constraint-based method (Zilles and Salisbury, 1995; Ruspini et al., 1997; Ortega, 2006; Ortega et al., 2007; Chan et al., 2016) were gradually developed to avoid penetration and to guarantee the smoothness of the output force.

Based on bone drilling simulation researches, many implant simulation methods involving haptics gradually come out (Ai et al., 2005; Kusumoto et al., 2010; Syllebranque and Duriez, 2010; Chen et al., 2012; Kinoshita et al., 2016; Pires et al., 2016). Chen et al. (2012) proposed a comprehensive preoperative planning and virtual training system on the basis of the Omega.6 haptic device and the CHAI3D toolkit. A similar implant simulator was the SimImplanto proposed by Pires et al. (2016), which uses Falcon to provide haptic feedback and controls the orientation through keyboard. These methods have little difference with bone drilling simulation methods. The main challenge of implantation simulation is the simulation of various motion constraints within the preparation bed. To make sure that a drill will not be able to enter a hole made by a smaller drill, Syllebranque et al. maintains a small enough global convergence criterion to keep precision in Syllebranque and Duriez (2010), which may affect the computation rate. Other constrains such as the axis constraint is not mentioned in their work. In Kinoshita et al. (2016), as the haptic device can only move along the single direction, the movement form of the tool is naturally restricted to one degree of freedom. The limitation of this method is that the free movement on bone surface will not be able to simulate, as well as the axis correction. To some extent, the axis-constrained movement within the preparation bed is similar to that of probing dental caries. Wang et al. (2016) simulated the insertion constraints using a virtual tunnel constraint. The method is not applicable to dental implantation as it only works on undestroyed surface and cannot distinguish predefined preparation beds effectively.

Generally the motion forms of an implant tool can be summarized as two categories, namely the high degree of freedom (H-DoF) motion during which the tool can move freely on bone surface and in free space with six degrees of freedom, as well as the low degree of freedom (L-DoF) motion during which the tool moves within the implant bed with restrained degrees of freedom. Mostly the tools would move along the bed axis through the implant duration due to their weak lateral cutting abilities. And some special constraint forms such as screwing are essential to the placement of the implant. Despite the importance of motion constraints for dental implantation simulation, up to now research works are mainly about the theories of bone drilling. In contrast to previous approaches, our work aims to simulate the complete dental implantation procedures involving diverse tool-tissue contact constraints through a state switching framework based on the haptic rendering methods of virtual coupling and virtual constraint. The contribution of this paper can be summarized as:

- A state switching framework for the complete process simulation of dental implantation. By analyzing the motion states of different tools, we divide the motion forms of the implant tools into two categories, i.e., the H-DoF motion of the dental tool probing against the teeth surface, as well as the L-DoF motion of the dental tool within the preparation bed. The framework can switch naturally between the two states according to the proposed implant criteria. During the whole workflow, the 1 kHz update rate can be maintained for stable haptic rendering.
- A virtual constraint haptic rendering method for simulating the constrained movement of the dental tool within the preparation bed, including sliding, drilling, screwing and perforating. Compared to H-DoF motion on bone surface and in free space, the constrained motion along the preparation bed axis has more subforms, among which some forms such as screwing are little mentioned in previous works. The virtual constraint method consists of three core steps, namely defining the movement axis, projecting the configuration difference and deriving the movement control ratio. Experimental results validate that the method can present all these motions precisely, and are capable to different tools.
- A two layered volumetric model for ensuring stability and efficiency. The simulation models of the tools and the bone are both the volume model due to its efficiency in collision detection and material removal. The tool volume is divided into the outer layer and the inner layer to indicate the interaction state, as well as to maintain the force stability and to accelerate collision detection.

Till now there are five kinds of simulation methods that are applicable to implant simulation or similar tasks, namely the penetration methods, the virtual constraint methods, the physical constraint methods that introduces the customized haptic device to provide physical constraints, the state-switching method for insertion simulation and our framework. The utilities of our framework and other rendering methods in implant simulation are summarized in Table 1. Compared with other work, the novelty and superiority of the rendering framework put forward by us lie in that it is applicable to different kinds of constraints. And for some procedures require simulation precision, our methods are able to reach high simulation precision as it utilizes geometric constraint.

**Table 1:** Utilities of different rendering methods in implant simulation.

	**Penetration methods**	**Constraint methods**	**Physical constraint methods**	**State-switching method**	**Our work**
6 DoF	Yes	Yes	No	Yes	Yes
Little penetration	No	Yes	Yes	Yes	Yes
Different tools	Yes	Yes	No	Yes	Yes
Sliding simulation	Yes	Yes	No	Yes	Yes
Drilling simulation	Yes	Yes	Yes	No	Yes
Insertion simulation	Yes	Yes	Yes	Yes	Yes
Insertion precision	Low	Middle	High	NA	High
Screwing simulation	No	No	No	No	Yes

The remainder of this paper is organized as follows. In section Analysis of the Dental Implant Procedures, we introduce the complete procedures of dental implantation briefly. In section Materials and Methods, we present the state switching framework for the haptic simulation of different motion forms, and the virtual constraint method for the L-DoF motion is explained in detail. Corresponding methods including the material removal and graphically rendering algorithms, the modeling methods of the alveolar bone and the implant tools, and the collision detection method are also contained in this section. Section Experiments displays the experiment results to validate the fidelity of the proposed methods, including force signal analysis and preliminary user studies. Finally, the conclusion and future work are discussed in Section Conclusions and Future Work.

## Analysis of the Dental Implant Procedures

**Figure 1:**
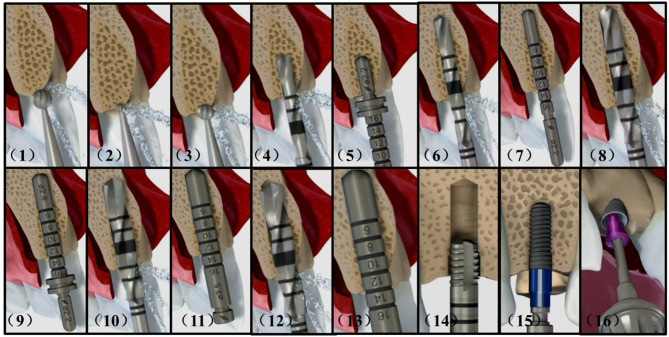
Complete implant procedures of the Straumann® BL Φ4.8–L12 mm RC. (1) smooth a narrow tapering ridge with the Ø 3.3 mm round bur (2) mark the implantation site with the Ø 1.4 mm round bur (3) widen the position of the mark with the Ø 2.3 mm round bur (4) mark the implant axis by drilling to a depth of about 6.0 mm with the Ø 2.2 mm pilot drill (5) insert the short side of the Ø 2.8 mm depth gauge to check for correct implant axis orientation (6) pre-drill the implant bed to about 12.0 mm with the Ø 2.2 mm pilot drill (7) use the Ø 2.2 mm depth gauge to check the implant axis and preparation depth (8) widen the implant bed with the Ø 2.8 mm pilot drill (9) check the preparation depth with the Ø 2.8 mm depth gauge (10) widen the implant bed to Ø 3.5 mm (11) check the preparation depth with the Ø 3.5 mm depth gauge (12) widen the implant bed with the Ø 4.2 mm pilot drill (13) check the preparation depth with the Ø 4.2 mm depth gauge (14) tapping the thread in dense bone (15) insert the implant with handpiece (16) close the implant with a closure screw.

Dental implantation surgery mainly involves bone drilling to prepare the implantation bed for placing the implant, and the operation procedures of different implant systems can be divided into similar steps. Figure 1 shows the complete procedures of the Straumann® BL Φ4.8 mm–L12 mm RC.

After opening the gingiva, the dentists firstly reduce and smooth a narrow tapering ridge to provide a flat bone surface. Then they mark the implantation site and the implant axis in sequence. It is necessary to check for correct implant axis orientation with the depth gauge or similar tools. The depth and radius of the implant bed will be widened gradually according to the type of the selected implant. Sometimes tapping is recommended in dense bone to achieve optimal primary stability before screwing the implant. When the above preparations are finished, the implant can be placed into the implantation bed. Finally the implant is closed with a closure screw or a healing cap.

During the above procedures, the biggest difference with ordinary bone drilling operations arise from the weak lateral cutting abilities of the implantation tools. The movement of the tool within the implant bed is highly restricted and is mostly restrained to moving along the bed axis. And the constrained motion could appear in different forms including sliding, drilling, screwing, and perforating according to tools types and motion conditions.

## Materials and Methods

### Simulation Models

#### Modeling of the Alveolar Bones

**Figure 2:**
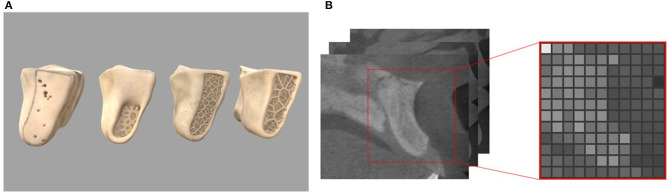
Four classes of the alveolar bone and read gray values from CT slices **(A)** geometric models of different bone types **(B)** CTCT slices and corresponding volume slice.

Implantation is performed mainly on the alveolar bone, which is composed of multi-layer materials with different properties. As shown in Figure 2, the outer layer of alveolar bone is compact bone with high hardness, and the inner layer is cancellous bone with relative low hardness. And according to the agenda and age of different people, the alveolar bone can be divided into four different types as class I, II, III, and IV. The hardness decreases with the increment of the class value.

In our work, we choose the volume model to represent the alveolar bone as it can store the physical properties and accurately record the topological change information. There are many tiny protrusions and depressions on the surface of the alveolar bone. In order to accurately describe these features, it is suggested to set the resolution of the volume to 128 or larger. The raw data used to construct the bone volume model is from the Cone Beam CT data of real patients, and is processed using Materialise's Mimics software to export the gray value data representing the bone density. After segmentation, a three-dimensional array representation storing attributes for each voxel is constructed. The subscript of the array corresponds to the coordinate relationship, and the value of the array represents the attribute of the voxel.

#### Modeling of the Tools

The tools are also described by the volume model in our work. The collision detection between two volume models can be quickly implemented by iterating the voxels on either of them. Since the voxel number of the alveolar bone is much larger than that of the tools, we prefer to iterate the voxels of the tools to improve the efficiency of collision tests. To maintain the fidelity of material removal, we always set the voxel size of the tool smaller than that of the alveolar bone. Then the volume model of the tool can be derived by measuring its geometric parameters.

**Figure 3:**
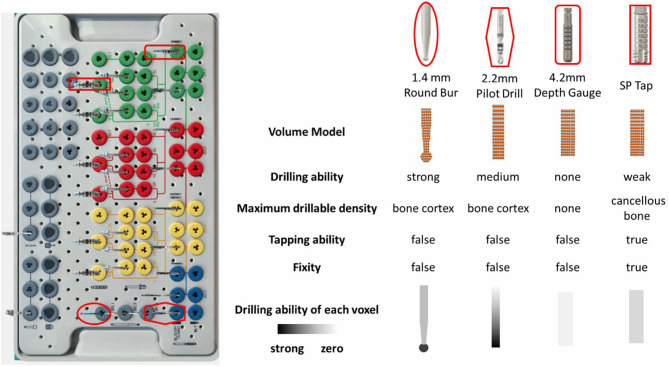
The typical tool kit of the Straumann® Dental Implant System and the property diagram of representative tools.

Nowadays there are hundreds of implant manufactures, and most manufactures provide the tool kits of their own for implementing surgeries to obtain ideal results. The number of tools in different tool kits ranges from several to dozens. Figure 3 shows the newest implant kit of the Straumann planting system, providing approximately fifty kinds of tools. In order to realistically redisplay the behaviors of various tools, the physical attributes are attached to the volume model and the individual voxel. The crucial physical attributes are summarized as follows:

- Drilling ability. This parameter directly determines whether the tool can remove material or not. Tools without the ability, such as the depth gauge, are not suitable for drilling usage.
- Maximum drillable density. If the contacting bone density exceeds the drillable density of the tool, the drill will be unable to work unless the existence of predefine preparation bed. And the tool will stop drilling immediately touching the bottom of the bed.
- Tapping ability. When there exists predefined preparation bed, tools with tapping ability can be screwed into the bed even if their diameters are a little larger than the bed diameter. And with the increment of advancing distance, it will become more and more difficult to pull the tool out.
- Fixity. Tools with tapping ability can obtain fixity if they are screwed in the smaller diameter implant bed. Thus, they become part of the jawbone and may collide with the newly selected tool.
- Drilling ability of each voxel. The drilling ability are not equally distributed on the volume. For example, the voxels on the lateral usually have little drilling abilities.

It should be noted that the above physical parameters are related to many other parameters, such as the rotation speed of the handpiece.

### Haptic Rendering

#### Overview of the Haptic Rendering Method

Despite the enormous quantity of the implantation tools and the wide variations among them, we manage to find out the simulation methods applicable to different tools. Compared with tools designed for dental restoration operations, the implantation tools show significant weakness on lateral cutting abilities. The interaction between implant tools and alveolar bone can be classified as two categories, which are L-DoF motion on bone surface and H-DoF motion within the implant bed.

Under the H-DoF motion state, the tool can slide freely on the surface of alveolar bone with six degrees of freedom. It mainly happens before the tool inserting into the implant bed. For tools with strong cutting abilities, such as round drills, the degree of motion freedom can be maintained even when the tool move within the implant bed.

Most operations during the implant procedure are carried out under the L-DoF motion when the tools have been inserted into the plantation bed. Unless the radius of the tool is much smaller than that of the bed, the tool are usually restricted to moving along the long axis of the bed. The possible motion forms of the tools include sliding, drilling, screwing and perforating.

- Sliding happens when the tool is partly inside the preparation bed and is sliding along and rotating about the long axis of the bed. The tool stops moving when it touches the bottom of the bed, while it can freely slide along the opposite direction until it totally separates from the bed.
- Drilling refers to the operation of expanding the depth or the diameter of the implant bed when touching the bed bottom. Some tools are specifically designed for removing the surrounding materials of the implant bed to expand the radius. For these tools, bed bottom refers to the highest bed surface of which the radius is smaller than the tool radius. During the advancing process, the tool gradually expands the bed radius until it touches the original bottom. As the center parts of the original bottom are undamaged, the tool can no longer move ahead.
- Perforating. Sometimes it is possible to drill through the alveolar bone with the tool tip outside the bone and the main body of tool inside the bone. Although the tool is still restricted to move along the bed axis, it can suddenly push ahead a certain distance. The user can evidently feel the sudden falling through of feedback force.
- Screwing. As for tools with tapping ability, they can be screwed into the implant bed even if their diameters are a little larger than the beds'. These kinds of tools can move within the implant bed only when the handpiece rotates clockwise for advancing and counterclockwise for stepping back.

**Figure 4:**
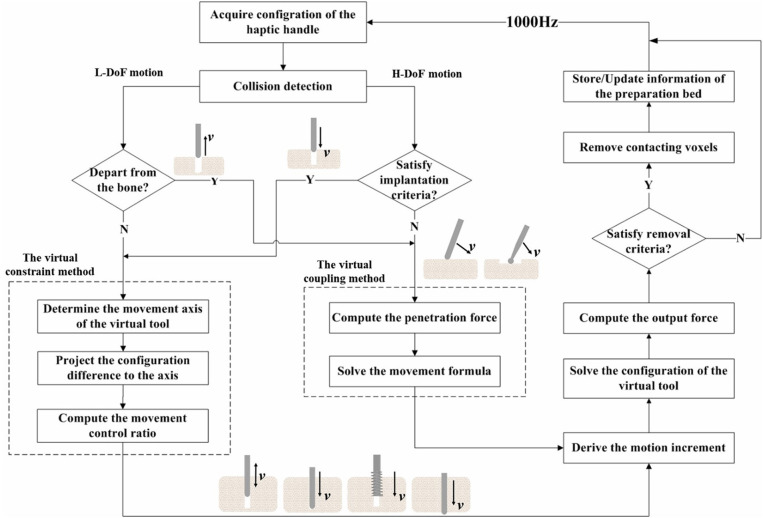
Flowchart of the state switching method.

Dealing with the various motion forms of the implant tools, we propose a state switching framework to simplify the simulation workload by rendering the H-DoF motion state and the L-DoF motion state separately, and seamless switch between the two states by defining an implant criteria as the switching judgment. In addition, we come up with the virtual constraint method to render the L-DoF motion, which shows efficiency and accuracy in adapting to different kinds of constraint forms. The H-DoF motion on bone surface and in free space is simulated through the previously proposed virtual coupling method (Ge et al., 2010). The detailed work flow of the framework is shown in Figure 4, where ***v*** represents the velocity of the haptic handle.

**Figure 5:**
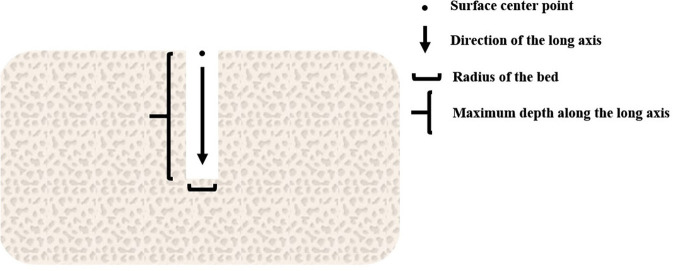
Information of the preparation bed.

Implant criteria are defined to trigger the switch to the constrained motion state, including (1) the tip of the bur coincide with the surface center point of the predefined implant bed, (2) the radius of the bur should be the same with or a little smaller than the radius of the contacting bed, (3) the axis of the bur must be aligned with the long axis of the bed, (4) the insertion force along the axis is greater than a pre-defined threshold. It should be noted that as long as the criterion (4) is satisfied, burs that can drill through the cortical bone will switch into the implant state when contacting the jawbone on the tip. When the bur creates a new hole on the surface of the jawbone, the information of the bed should be stored in a carefully maintained list for possible later matching. As is shown in Figure 5, the information includes the axis direction, the tip point and the radius of the implant bed.

In the following Sections The Virtual Coupling Method and The Virtual Constraint Method, the methods for simulating the two stages will be explained, and we focus on the virtual constrained method.

#### The Virtual Coupling Method

In our previous work (Ge et al., 2010), we introduced a virtual coupling method to solve the interaction between the volume models. The method computes the penetration force ***F***_***G***_ first. And by adding a virtual spring between the virtual tool and the haptic tool, it solves the configuration of the virtual tool. The output force ***F*** can be finally derived using the following equation, in which *k* is the stiffness of the haptic device, ***p***_*Handle*_ is the position of the haptic handle, and ***p***_*Bur*_ is the position of the virtual tool.

(1) $$\begin{array}{r} F\;=\;k\;\cdot \left(p_{Bur}-p_{Handle}\right) \end{array}$$

The penetration force are obtained by integrating the unit force acting on the outer voxels of the tool. The value of the unit force is non-zero only when the corresponding tool voxel embedded into the bone. The calculation formula is as follows:

(2) $$\begin{array}{r} F_{G}\;=\;\frac{1}{2\pi N}\iint_{D}u\left(s\right)v\cdot n_{n}(s)\frac{1}{r\left(s\right)}[-\frac{1}{\alpha }n_{n}\left(s\right)\;+\;n_{t}(s)]ds \end{array}$$

Where ***v*** is the translation speed of the bur, *ds* is the contact area, *r*(*s*) is the radius of the tool, *N* is the rotation speed, ***n***_***n***_(*s*) and ***n***_***t***_(*s*) are the normal and tangent vectors of the tool, respectively, α is the proportional coefficient of the normal force and the tangential force, *u*(*s*) is the cutting factor of the material. α and *u*(*s*) are related to the properties of the material and the bur, and can be measured experimentally.

**Figure 6:**
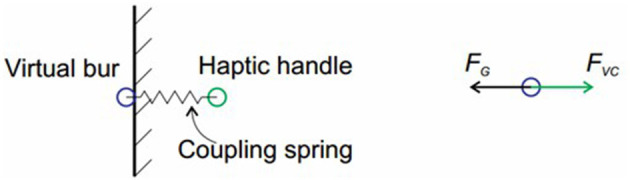
Illustration of the virtual coupling method.

In order to maintain the smoothness of the output force and reduce the penetration between tools and bone, the virtual coupling method is adopted. This method introduces the virtual tool into the system as the avatar of the haptic handle. It is shown in Figure 6 that the virtual tool is connected with the haptic handle through the virtual spring, and its position is decided by both the penetration force ***F***_***G***_ and the virtual coupling force ***F***_***vc***_. The virtual spring force is calculated as:

(3) $$\begin{array}{r} F_{vc}\;=\;k_{vc}\left(p_{Handle}\;-\;p_{Bur}\right) \end{array}$$

Where *k*_*vc*_ is the stiffness coefficient of the virtual spring, ***p***_***Handle***_ is the position of the haptic handle, and ***p***_*Bur*_ is the position of the virtual tool. The state of the virtual tool can be represented as:

(4) $$\begin{array}{r} y\left(t\right)={\left(p_{bur},\;v\right)}^{T} \end{array}$$

Assume that ***v*** is the velocity of the tool and *m* is the mass of the tool, the movement of the virtual tool can be described by the following first-order differential equation:

(5) $$\begin{array}{r} \overset{∙}{y}\left(t\right)\;=\;{\left({\overset{∙}{p}}_{Bur},\;\overset{∙}{v}\right)}^{T}={\left(v\frac{\left(F_{vc}\;+\;F_{G}\right)}{m}\right)}^{T} \end{array}$$

Using the implicit integral method and the Gaussian linear elimination method to solve the above formula, the configuration increment Δ***p***_*bur*_ of the virtual tool can be obtained. Finally the next configuration of the virtual tool $p_{bur}^{t+1}$ can be solved through adding the increment to ***p***_*bur*_.

#### The Virtual Constraint Method

To deal with the various motion forms of the tools in the L-DoF motion stage, we propose a virtual constraint method. The utility of the method is proved by experiments in Section Experiments. As is shown in Figure 5, the method consists of three core procedures to derive the configuration of the virtual tool, including defining the movement axis of the tool, projecting the configuration difference to the axis and calculating the movement control ratio.

The virtual constraint in this paper mainly refers to the axial restraint acting on the implant tools, which is imposed by the implantation bed. Under the constraint, the tool is restricted to moving along and rotating about the long axis of the bed. For tools that can drill through the bone surface, the implant bed and the axial constraint can be created by themselves. While for tools that have weak cutting abilities, the constraint usually arises from the predefined implant beds created by other tools.

When the tool enters the constrained motion mode, we also connect the haptic handle with the virtual tool through the virtual spring, and calculate the output force with Equation (1). Considering the anisotropy of the bone density inside the alveolar bone, *k* is no long a constant value, but calculated by weighting the gray value of the bone voxels through the contact area with the tool tip:

(6) $$\begin{array}{r} k=\underset{i}{\sum }w_{i}\cdot gray_{i} \end{array}$$

Where *w*_*i*_ is dependent on the embedded area between the voxels, and *gray*_*i*_ is the gray value of the bone voxel.

Through the L-DoF motion period, the motion axis of the tools is always definite. Thus the configuration increment Δ***p***_*bur*_ of the virtual tool can be mapped directly from the configuration difference of the haptic handle and the current virtual tool. The virtual constraint method starts by projecting the configuration difference between the virtual tool and the haptic handle to the long axis:

(7) $$\begin{array}{l} \Delta y_{p}=\;\{\begin{array}{c} \;0\;num_{cd}==0\\ \left(\Delta p\cdot n_{hole}\right)\;\cdot \;n_{hole}\;otherwise \end{array} \end{array}$$

(8) $$\begin{array}{r} \Delta p\;=\;\;p_{Handle}-p_{Bur} \end{array}$$

**Figure 7:**
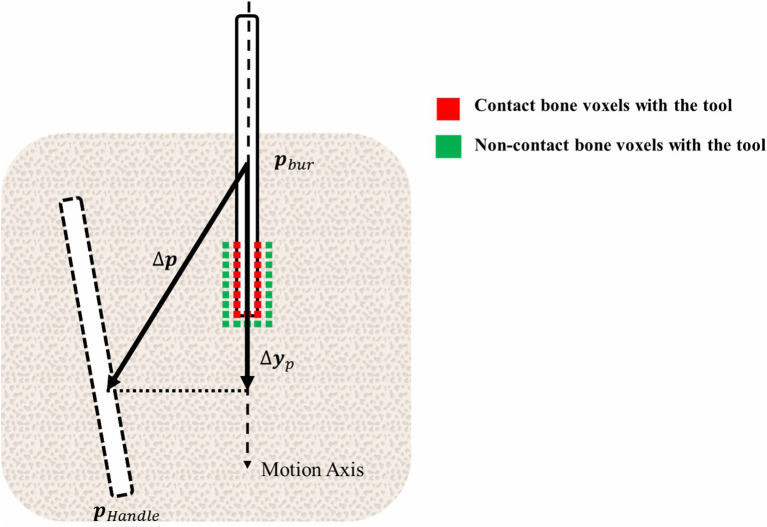
Illustration of the virtual constraint method.

Where ***n***_*hole*_ is the unit normal vector of the implant bed long axis, pointing from bone surface to the implant bed bottom, and **Δ*p*** represents the configuration difference between the haptic handle and the virtual tool. Δ***p*·*n***_*hole*_, the projection of the position difference on the insertion axis, indicates the motion trend with its positive value represents advancement and negative value represents pulling out. *num*_*cd*_ is the number of bone voxels colliding with the tool voxels currently. If the value of *num*_*cd*_ is not zero, it means that there are obstacles in advance. And thus the tool is not allowed to push ahead until it removes all these obstacles. Of course, the tool will suffer new obstacles in the next position. The obstacles here refer to the voxels of the bone. Equations 7, 8 are illustrated in Figure 7.

**Figure 8:**
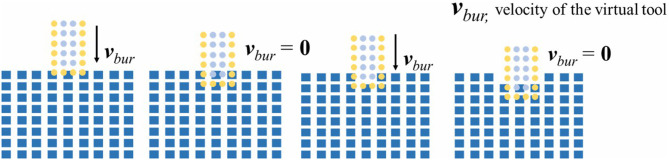
The two layer model for bone dissection.

It should be noted that either when the tool clears the contacting bone voxels or when the tool totally separates from the bone, the value of *num*_*cd*_ can be zero. This ambiguity brings problem to using *num*_*cd*_ as the judgment basis of progression. Fortunately the previous proposed two-layer volume model (Ge et al., 2010) is suitable for solving this problem. As is illustrated in Figure 8, voxels on tool volume are divided into the 1~2 layer outer voxels and the remaining inner voxels. We count the collided tools voxels with bone, respectively, for the outer voxels and the inner voxels. And the variable *num*_*cd*_ is specified as the number of the inner voxels. In addition, the variable *num*_*out*_ represents the number of collided outer voxels. Obviously when both variable values are zero, the tools would apart from the bone. And if only *num*_*cd*_ is zero, it demonstrates that the tools under the L-DoF motion state has cleared all the obstacles.

In practice, the motion range of the dentists seldom go beyond the size of the single voxel within 1 ms. The Equation (7) has the potential to cause the sudden coincidence of the virtual tool to the haptic handle, which brings force discontinuity. In order to prevent the discontinuity, we limit the moving distance of the virtual tool in a single cycle:

(9) $$\begin{array}{r} \Delta y_{c}=\;\{\begin{array}{c} \;\Delta y_{p}\;|\;\Delta y_{p}|<step\;\\ step\cdot \frac{\;\Delta y_{p}}{\;\Delta y_{p}}\;otherwise \end{array} \end{array}$$

In which *step* refers to the size of a single voxel.

As demonstrated by equation 10, the configuration increment of the virtual tool Δ***p***_*bur*_ can be finally derived by multiplying the limited moving distance Δ***y***_***c***_ with the movement control ratio *w*. The movement control ratio ranges from 0 to 1, and it plays the vital role in distinguishing among different constrained motion forms.

(10) $$\begin{array}{r} \Delta p_{bur}\;=\;w\cdot \Delta y_{c} \end{array}$$

When the tools do not reach the bottom of the implant bed, it can freely slide along the axis. The friction resistance can be simulated by adjusting the value of *w*. When the tool reaches the bottom, it is not allowed to move ahead as there are obstacles. Even if the value of *w* is not changed, the movement will slow down as it takes time to remove contacting obstacles. However, the tool can still be pulled out freely as the motion obstruction disappears in the opposite direction. One exception is that if the tool has tapping ability, it can be tightly located in the implantation bed, being unable to be pulled out. During the taping operation, the value of *w* is switched between zero and non-zero value according to many factors, including the applied force, the rotation speed and orientation of the handpiece, and the movement trend of the haptic tool. After perforation, the value of collided voxels number *num*_*cd*_ is always zero. As the voxel step value in Equation (9) is much larger than the single-cycle movement range of the haptic handle, the virtual tool can catch up with the haptic handle after very few cycles, namely instantly, to cause the sudden force falling through.

In summary, all the constraint forms can be simulated through the virtual constraint method, validating the applicability and generality of the method.

### Dissection Simulation

In the real world, two solid substances like the alveolar bone and the implant tool will never occupy the same place. Thus, when collision happens between the voxels of bone and the tool voxels, the embedded part on the bone can be drilled away. However, as the single-cycle movement distance of the virtual tool is normally smaller than the bone voxel size, the tool will lose contact with the bone if all embedded bone voxels are erased until it retouches the bone in certain cycles. This kind of frequent switching between the contact state and the separation state can easily cause discontinuities to feedback force.

To address the problem, we only remove the bone volume voxels collided with the inner tool voxels in practice. As is shown in Figure 8, this method maintains the contact between the outer 1~2 layer tool voxels and the bone voxels. Because the single-cycle moving distance of the tool is much smaller than the size of the voxel, we use only the outermost n-layer inner voxels of the tool for cutting calculation, *n* is defined as:

(11) $$\begin{array}{r} n\;=\left|{\Delta p}_{Bur}\right|/step \end{array}$$

**Figure 9:**
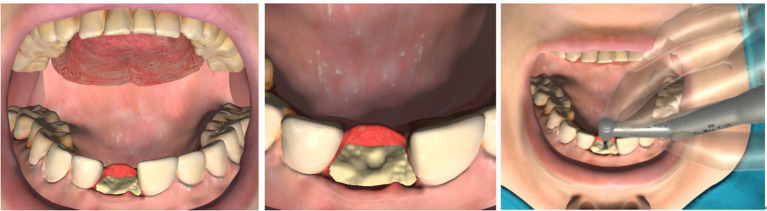
The interactive scenes between the tools and bone of different stages.

During the drilling process, we need to refresh the triangle meshes of the bone according to the topological changes. The refresh frame rate should be higher than 30 Hz for good visual effects. The reconstruction method used in our work is the Marching Cube (MC) Method. The outstanding characteristic of this method is that it allows us to divide the voxel model into different subblocks. Each of the subblcoks can be drawn independently. This feature makes the method suitable for GPU acceleration and convenient for us to pick up the destroyed subparts to redraw. The interactive scenes between the tools and bone of different stages are shown in Figure 9.

## Experiments

**Figure 10:**
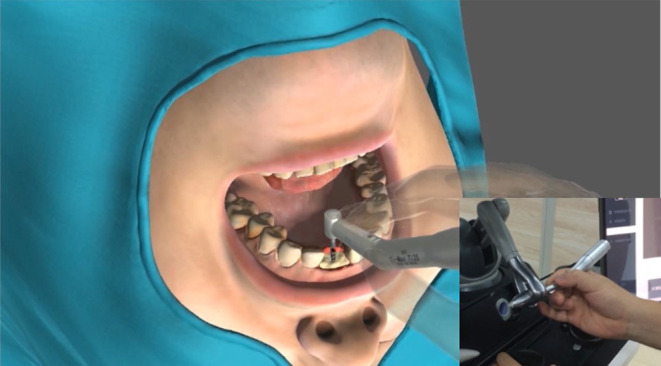
The haptic system of the experiments.

In this section, we present evaluation experiments to validate whether the proposed approach can simulate various implant procedures. We first analyze the force signals under different implant scenarios, and then we present a preliminary user study to validate system realism. The haptic system of the experiments is composed of an implantology handpiece fixed on a Phantom Omni device. And the virtual environment includes a volumetric jawbone, the entire set of Straumann implant tools, and the virtual patient, as is shown in Figure 10. In our experiments, the sixteen complete implantation procedures of the Straumann® BL Φ4.8 mm–L12 mm RC are carried out. The Supplementary Video 1 displayed the complete process, and the values of relative parameters are listied in Table 2.

**Table 2:** The value of some key parameters.

**Properties**	**Values**
Volume size	20 mm * 18 mm * 8 mm
Volume resolution	256 *128 * 128
Average stiffness of cancellous bone	4.0 NS/mm
Average stiffness of compact bone	1.0 Ns/mm
Update rates	>1,000 Hz
Average drilling speed of sphere burs	2.0 mm/ms
Maximum drilling stiffness of round burs	5.0 Ns/mm
Average drilling pilot drills	1.0 mm/ms
Maximum drilling stiffness of pilot drills	5.0 Ns/mm
Average drilling speed of twist drills	0.7 mm/ms
Maximum drilling stiffness of twist drills	2.0 Ns/mm

**Figure 11:**
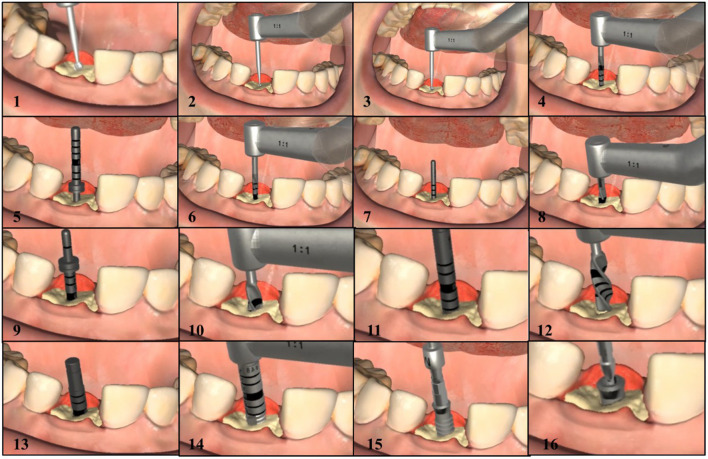
The screenshots of the complete implant procedures.

The reason we choose Straumann® Dental Implant System for testing is that it is one of the most widely used implant systems worldwide, and its procedures are fairly typical. Figure 11 shows the screenshots of the complete implant procedures.

**Figure 12:**
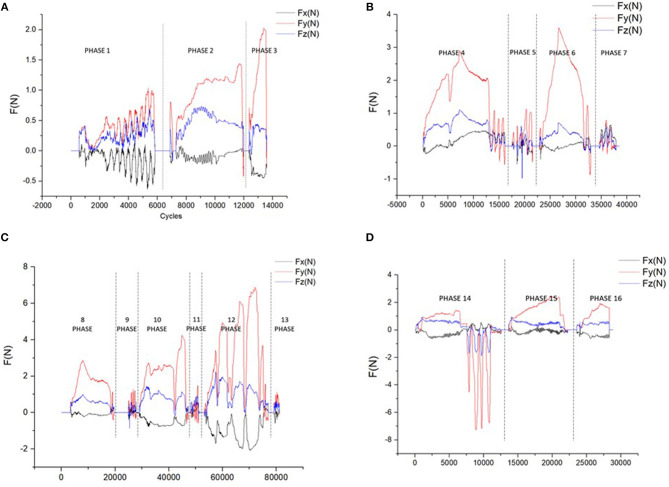
The force curve of different phases **(A)** curves of the 3.3/1.4/2.3 mm round burs **(B)** curves of the 2.2 mm pilot drills and depth gauges **(C)** curves of the 2.8/3.5/4.2 mm twist drills and depth gauges **(D)** curves of the tap, implant, and the closure screw.

Figure 12 shows the force curve obtained during the implant process of the Straumann® BL Φ4.8 mm–L12 mm RC. The whole process consists of 16 different phases. As the output force is a 3-DoF vector, we use its components Fx, Fy, and Fz along the x-axis, y-axis, and z-axis to represent it. In our work, the force coordinate system coincide with the screen coordinate system, with the positive x direction toward right, the positive y direction toward upward and the positive z direction opposite to the line of sight direction.

Figure 12A shows the curves of the round burs with different diameters of 3.3, 1.4, and 2.3 mm. Phase 1 show the curve during smoothing the narrow tapering ridge, and phase 2 and phase 3 shows the force curve of marking the implantation site. The smaller difference on the magnitudes of different force components in phase 1 indicates that the tool is sliding freely on the surface, while in phase 2 and phase 3 the y-axis component gradually increases as the drilling direction is nearly downward.

The force curve of the 2.2 mm pilot drill and depth gauge are shown in Figure 12B. The 2.2 mm pilot drill firstly marks the implant axis by drilling to a depth of about 6.0 mm (phase 4). Then we insert the depth gauge to check the implant axis orientation (phase 5). After examination the implant bed is drilled to the final preparation depth with the 2.2 mm pilot drill (phase 6), and the depth gauge is used to check the orientation again (phase 7). Figure 12B indicates that the y-axis force component holds the dominant role during the drilling process, indicating the axis orientation is nearly downward. As the drilling depth is prepared to 6.0 mm in phase 4, the tool can slide along the bed until it touches the bottom. This can be reflected by the low force curve in phase 6 and phase 7, as well as the rapid rise of y-force component at the beginning of phase 6.

As is illustrated in Figure 12C, the 2.8/3.5/4.2 mm twist drills are used in sequence to widen the implant bed (phase 8, phase 10, phase 12), and the corresponding depth gauges are used to check the orientation after drilling (phase 9, phase 11, phase 13). It can be seen from the force curve that it requires bigger force to drill the contacting voxels with the increase of the diameter. During the drilling process, the tool needs to be pulled back and forth to dissipate heat, which can be demonstrated by the rapid force changes in phase 8/10/12, and is most evident in phase 12.

Figure 12D shows the force curve during the placement of the implant (phase 15) and the closure screw (phase 14). The entire length of the implant bed needs to be tapped with the tap (phase 13) before the placement of the implant. The tools used in the three phases have tapping ability, and thus cannot be pulled out freely as the former tools. In phase 14, we tried to pull the tap out without adjusting the rotation clock direction of the handpiece but failed, leading to the large resistance force in opposite direction.

**Figure 13:**
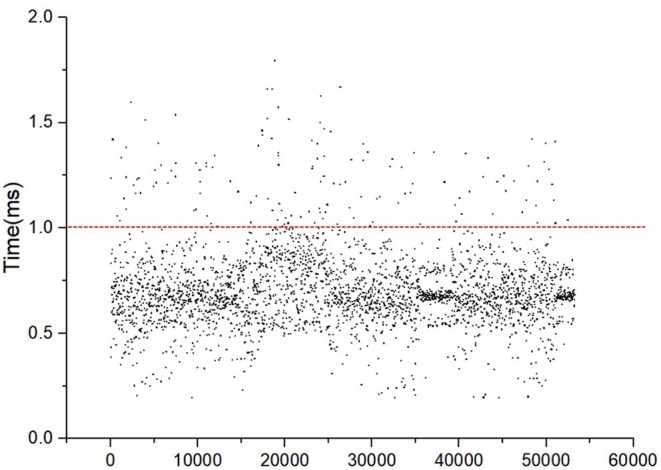
The force synthesis time clouds.

Figure 13 shows the force-synthesis time point clouds obtained in the implant process of the Straumann® BL Φ4.8 mm–L12 mm RC. In Figure 13, the x axis represents the serial number of the force refresh cycles, and the y axis represents the total refresh time per cycle, namely the sum of time spending on posture inquiry, collision detection, collision response, force output and so on. It is evident that the refresh time is <1 ms under most circumstances. In other words, the refresh rate can be up to more than 1,000 Hz.

In order to verify the validity of the algorithm, we organized some preliminary tests among dentists. Most experts gave active feedback on haptic-based virtually surgery training. Experts spoke highly of the force perception. They said that the training system can simulate the feelings of different parts of the alveolar bone by evidently distinguishing between cortical bone and cancellous bone. During the implant procedures, the system could also simulate the constraints imposed on various planting tools by the alveolar bone with great reality. And unpleasant phenomena such as that large diameter drills can be inserted into small diameter holes didn't occur. The feeling of force falling through when drilling through the bone was also obvious. However, the experts suggested that the virtual environment could be more realistic if the real-time deformation of soft tissues could be introduced.

The above results show that the dental implant algorithm based on the state switching framework can effectively realize force feedback of the implantation process.

## Conclusions and Future Work

To achieve the simulation of the complete implant procedures, the realistic simulation of the constrained movement of different implant tools and natural switching between the free motion on the surface and the constrained motion within the preparation bed are fundamental requirements. In this paper, we propose a state switching framework to seamless switch between the free motion state and the constrained motion state. Free motion on the surface can be simulated through the previous proposed virtual coupling method. And the virtual constraint method is built up to render the constrained motion, which shows efficiency in adapting to different kinds of constraint forms during the procedures of sliding, pulling, screwing, and perforating. Experimental results based on a Phantom Omni device illustrated that the proposed method could simulate the complete implant procedures of Straumann® BL Φ4.8 mm–L12 mm RC, which consists of 16 different phases. According to the output force curve, different constraint forms could be presented with steady and continuous output force during the operation procedures. And the force features of different constraints are analyzed through the force curve. Preliminary user studies have shown that the virtual perception matches with that of the clinical practice.

In order to physically validate the realism of the proposed rendering methods, one possible future Research Topic is to measure the interaction force applied on the implant tools during clinical operations, along with the measurement of the trajectory and velocity of the moving tools. These measured data could possibly provide ground truth to evaluate the proposed haptic rendering algorithm. In the next step, we also plan to simulate the deformation behavior of the gingival and other deformable tissues. This can not only improve the realism of the virtual environment, but also be helpful to simulation of the preoperative procedures such as opening the gingival, which are vital to the success of implant surgeries and thus need to be trained. Furthermore, the methods should be employed on different kinds of implant systems. There are hundreds of implant brands nowadays. While the dentists may use more than one kind of systems in practice, they normally practice on the same system in school. Therefore, it is meaningful to find the simulation methods applicable to most systems to provide the dentists the opportunities to get familiar with operation procedures of different implant systems. With the above fundamental work finished, it is our final goal to construct the implant simulators for training dental school students, and carry out user studies to validate the significance of virtual training in implant skills education.

## Data Availability Statement

All datasets generated for this study are included in the article/Supplementary Material.

## Author Contributions

ZZ put forward requirements on the algorithm and collected CBCT data. XZ wrote the paper, proposed the algorithm and implemented it. DW, YC, and YZhao updated the algorithm. DW, YZhan, and ZZ revised the paper.

### Conflict of Interest

The authors declare that the research was conducted in the absence of any commercial or financial relationships that could be construed as a potential conflict of interest.

## Supplementary Material

Supplementary Video 1 The implantation operation video that shows the complete operation process of the virtual reality dental implantation.

## References

[B1] Acosta E. Liu A. (2007). “Real-time volumetric haptic and visual burrhole simulation,” in Virtual Reality Conference VR '07 (Charlotte, NC: IEEE), 247–250. 10.1109/VR.2007.352492

[B2] Agus M. Giachetti A. Gobbetti E. Zanetti G. Zorcolo A. (2003). Real-time haptic and visual simulation of bone dissection. Presence: Teleoper. Virtual Environ. 12, 110–122. 10.1162/105474603763835378

[B3] Ai Z. Evenhouse R. Rasmussen M. (2005). Haptic rendering of volumetric data for cranial implant modeling. Conf. Proc. IEEE Eng. Med. Biol. Soc. 5, 5124–5127. 10.1109/IEMBS.2005.1615630 17281400

[B4] Avila R. S. Sobierajski L. M. (1996). “Haptic interaction method for volume visualization,” in Visualization '96 Proceedings (San Francisco, CA).

[B5] Chan S. Li P. Locketz G. Salisbury K. Blevins N. H. (2016). High-fidelity haptic and visual rendering for patient-specific simulation of temporal bone surgery. Comput. Assist. Surg. 21, 85–101. 10.1080/24699322.2016.1189966 27973948

[B6] Chen X. Lin Y. Wang C. Shen G. Wang X. A. (2012). Virtual training system using a force feedback haptic device for oral implantology. Lect. Notes Computer Sci. 232–240. 10.1007/978-3-642-31439-1_21

[B7] de Boer I. R. Bakker D. R. Wesselink P. R. Vervoorn J. M. (2012). The Simodont in dental education. Ned Tijdschr Tandheelkd 119, 294–300. 10.5177/ntvt.2012.06.12105 22812267

[B8] Duriez C. Dubois F. Kheddar A. Andriot C. (2008). Realistic haptic rendering of interacting deformable objects in virtual environments. IEEE Trans. Visual. Comp. Graph. 12, 36–47. 10.1109/TVCG.2006.13 16382606

[B9] Forsslund J. Sallnas E. Palmerius K. (2009). “A user-centered designed FOSS implementation of bone surgery simulations,” in Proceedings of World Haptics Conference (Washington, DC), 391–392. 10.1109/WHC.2009.4810916

[B10] Ge Y. Wang D. Zhang Y. (2010). Voxel-based modeling for haptic-enabled dental drilling simulation. Chin. High Technol. Lett. 20, 314–319. 10.1115/DETC2009-86661

[B11] Kim L. Park S. H. (2006). Haptic interaction and volume modeling techniques for realistic dental simulation. Visual Comput. 22, 90–98. 10.1007/s00371-006-0369-8

[B12] Kinoshita H. Nagahata M. Takano N. Takemoto S. Matsunaga S. Abe S.. Development of a drilling simulator for dental implant surgery. J. Dental Educ. (2016) 80, 83–90. Available online at: http://www.jdentaled.org/content/80/1/83. 26729688

[B13] Kusumoto N. Sohmura T. Yamada S. Wakabayashi K. Nakamura T. Yatani H. (2010). Application of virtual reality force feedback haptic device for oral implant surgery. Clin. Oral Implants Res. 17, 708–713. 10.1111/j.1600-0501.2006.01218.x 17092231

[B14] Luciano C. Banerjee P. Defanti T. (2009). Haptics-based virtual reality periodontal training simulator. Virt. Real. 13, 69–85. 10.1007/s10055-009-0112-7

[B15] Mcneely W. A. Puterbaugh K. D. Troy J. J. (1999). “Six degree-of-freedom haptic rendering using voxel sampling,” in Proceedings of the 26th Annual Conference on Computer Graphics and Interactive Techniques. Los Angeles, CA: ACM Press/Addison-Wesley Publishing Co 10.1145/311535.311600

[B16] Morris D. Sewell C. Barbagli F. Salisbury K. Blevins N. H. Girod S. (2006). Visuohaptic simulation of bone surgery for training and evaluation. IEEE Comput. Graph. Appl. 26, 48–57. 10.1109/MCG.2006.140 17120913

[B17] Ortega M. (2006). A six degree-of-freedom god-object method for haptic display of rigid bodies. Proc. IEEE Virt. Real. Conf. 13, 191–198. 17356213 10.1109/TVCG.2007.1028

[B18] Ortega M. Redon S. Coquillart S. (2007). A six degree-of-freedom god-object method for haptic display of rigid bodies with surface properties. IEEE Trans. Visual. Comp. Graph. 13, 458–469. 10.1109/TVCG.2007.1028 17356213

[B19] Petersik A. Pflesser B. Tiede U. Hoehne K. H. Leuwer R. (2002). “Haptic volume interaction with anatomic models at sub-voxel resolution” in HAPTICS 02: Proceedings of the 10th Symposium on Haptic Interfaces for Virtual Environment and Teleoperator Systems (Washington, DC), 66.

[B20] Pires L. A. Serpa Y. R. Rodrigues M. A. F. (2016). “SimImplanto - a virtual dental implant training simulator,” in 2016 XVIII Symposium on Virtual and Augmented Reality (SVR) (Gramado: IEEE). 10.1109/SVR.2016.41

[B21] Ranta J. F. Aviles W. A. (1999). “The virtual reality dental training system—Simulating dental procedures for the purpose of training dental students using haptics,” in Proceedings of 4th PHANTOM Users Group Workshop, (Dedham, MA) 67−71.

[B22] Ruspini D. C. Kolarov K. Khatib O. (1997). “The haptic display of complex graphical environments,” in Conference on Computer Graphics & Interactive Techniques (Los Angeles, CA). 10.1145/258734.258878

[B23] Syllebranque C. Duriez C. (2010). “Six degree-of freedom haptic rendering for dental implantology simulation,” in Proceedings of the 5th International Conference on Biomedical Simulation (Phoenix, AZ: Springer-Verlag). 10.1007/978-3-642-11615-5_15

[B24] Thomas G. Johnson L. Dow S. Stanford C. (2000). “The design and testing of a force feedback dental simulator,” in Commutating Methods Programs Biomed, Vol. 64, 53–64. 10.1016/S0169-2607(00)00089-4 11084233

[B25] Tse B. Harwin W. Barrow A. Quinn B. San Diego J. P. Cox M. J. (2010). “Design and development of a haptic dental training system – hapTEL,” in Haptics: Amsterdam: Generating & Perceiving Tangible Sensations, International Conference, Eurohaptics (DBLP) 10.1007/978-3-642-14075-4_15

[B26] Wang D. Zhao X. Shi Y. Zhang Y. Hou J. Xiao J. (2016). Six degree-of-freedom haptic simulation of probing dental caries within a narrow oral cavity. IEEE Trans. Haptics 9, 1–1. 10.1109/TOH.2016.2531660 26915130

[B27] Yamaguchi S. Yoshida Y. Noborio H. Murakami S. Imazato S. (2013). The usefulness of a haptic virtual reality simulator with repetitive training to teach caries removal and periodontal pocket probing skills. Dental Mater. J. 32, 847–852. 10.4012/dmj.2013-174 24088844

[B28] Zilles C. B. Salisbury J. K. (1995). “A constraint-based god-object method for haptic display,” in Proceedings 1995 IEEE/RSJ International Conference on Intelligent Robots and Systems (Pittsburgh, PA: Human Robot Interaction and Cooperative Robots).

